# Understanding others: a pilot investigation of cognitive and affective facets of social cognition in patients with 22q11.2 deletion syndrome (22q11DS)

**DOI:** 10.1186/s11689-017-9216-7

**Published:** 2017-09-25

**Authors:** D. Badoud, M. Schneider, S. Menghetti, B. Glaser, M. Debbané, S. Eliez

**Affiliations:** 1 Developmental Clinical Psychology Research Unit, Faculty of Psychology and Educational Sciences, Geneva, Switzerland; 2 Lab of Action and Body, Department of Psychology, Royal Holloway, University of London, Egham, UK; 3 Office Medico-Pédagogique Research Unit, Faculty of Medicine, University of Geneva, Geneva, Switzerland; 4 Center for Contextual Psychiatry, Research group psychiatry, Department of Neurosciences, KU Leuven, Leuven, Belgium; 5 Research Department of Clinical, Educational and Health Psychology, University College London, London, UK; 6 Department of Genetic Medicine and Development, Faculty of Medicine, University of Geneva, Geneva, Switzerland

**Keywords:** Social cognition, Emotion recognition, Perspective taking, Theory of mind, Social functioning, Psychosis, 22q11.2 deletion syndrome, Adolescence

## Abstract

**Background:**

Although significant impairments in the affective and cognitive facets of social cognition have been highlighted in patients with 22q11.2 deletion syndrome (22q11DS) in previous studies, these domains have never been investigated simultaneously within the same group of participants. Furthermore, despite theoretical evidence, associations between these two processes and schizotypal symptoms or social difficulties in this population have been scarcely examined.

**Methods:**

Twenty-nine participants with 22q11DS and 27 typically developing controls (*N* = 5 siblings; *N* = 22 unrelated controls) aged between 11 and 21 years participated in the study. Both groups were matched for age and gender distribution. Two computerized social cognition tasks evaluating perspective and emotion recognition abilities were administered to all participants. The levels of schizotypal trait expression and social functioning were further investigated in both groups, based on a validated self-report questionnaire (Schizotypal Personality Questionnaire) and parental interview (Vineland Adaptive Behavior Scales).

**Results:**

Participants with 22q11DS exhibited lower perspective-taking and emotion recognition capacities than typically developing controls. The two socio-cognitive dimensions investigated here were further correlated in healthy controls. The efficiency of perspective-taking processes (response time) was marginally related to the degree of schizotypal trait expression in patients with 22q11DS.

**Conclusions:**

This study first provides support for significant deficits in two core facets of social cognition in 22q11DS. The associations observed between the experimental tasks and measures of social functioning or schizotypal symptoms in 22q11DS open promising research avenue, which should be more deeply investigated in future studies.

## Background

Patients with 22q11.2 deletion syndrome (22q11DS), a frequent neurogenetic condition occurring in up to 1:1000 pregnancies [1], are characterized by an increased risk of developing schizophrenia in adolescence and early adulthood (i.e., up to 20% of risk [2]). Across a broader age range, individuals affected by this genetic condition also experience higher schizotypal traits [2], which are considered as one of the possible phenotypic expressions of latent vulnerability to psychosis (i.e., schizotypy) [3]. Furthermore, impaired functioning, especially in the social domain, constitutes one of the hallmarks of the 22q11DS clinical phenotype [4, 5]. A better understanding of the mechanisms underlying schizotypal traits and social functioning is crucial in order to improve clinical interventions in this population. Social cognition refers to the way people make sense of others’ behavior and has been conceptualized as a factor contributing to social impairments and psychotic symptoms in patients with schizophrenia (e.g., [6]). Social cognition is a multifaceted construct, in which affective (i.e., ability to perceive and understand others’ emotional states) and cognitive (i.e., ability to infer others’ beliefs and intentions or theory of mind (ToM) and its related processes such as perspective-taking) dimensions can be distinguished [7]. Although these abilities are conceptualized as distinct processes, they are not completely independent and interact in many ways to produce adapted social behaviors [8].

The affective dimension of social cognition has been particularly studied in the field of 22q11DS. Indeed, several studies found that patients with 22q11DS were impaired in their ability to recognize facial emotional expressions [5, 9–12], or infer emotions of characters presented in short stories [13]. Gur et al. recently showed that the affective facet of social cognition was more impaired than expected based on intellectual disability level and that it followed an atypical developmental trajectory over time [11]. In their study, the authors computed developmental charts for several cognitive domains including social cognition (composite measure covering emotion identification, emotion intensity differentiation, and age differentiation) using the performance of typically developing individuals aged 8 to 21 years as the normative group. The chronological age of patients with 22q11DS was then compared to a developmental age (reflecting the difference between the score obtained by the participants and the norm) computed for each cognitive domain. These analyses showed that patients with 22q11DS steadily lagged behind the normative group on the social cognition composite score after age 8, but the gap increased and widened after age 14. However, a recent study showed that the recognition of facial emotions was relatively unimpaired in individuals with 22q11DS compared to typically developing controls when dynamic pictures of faces were presented [14]. Shashi et al. also reported similar performance in the recognition of vocal emotional expressions compared to the control group [5]. Altogether, these findings suggest that individuals with 22q11DS may be characterized by alterations of the affective dimension of social cognition. In particular, this seems to be the case when it is assessed in the visual modality and using static stimuli, which could be related to the use of atypical gaze patterns during visual exploration [9, 14–17].

Similar to its affective counterpart, the cognitive facet of social cognition was also found to be impaired in children and adolescents with 22q11DS in studies [12, 18] that used ToM tasks such as the Awareness of Social Inference Test (TASIT; [19]) or the Animation Task (see [20]). However, one study using false-belief tasks observed that ToM abilities had a delayed development in 22q11DS rather than long-lasting deficits. According to Campbell et al., these incongruent findings may be due to the fact that false-belief tasks rely strongly on verbal comprehension, whereas the remaining ToM paradigms used visual material (sometimes in combination with verbal narratives) [13]. In addition, a ceiling effect in the control group for some of the false-belief tasks may have prevented the detection of significant group differences.

Although findings are generally indicative of significant impairments in the affective and cognitive facets of social cognition in patients with 22q11DS, these domains have never been investigated simultaneously within the same group of participants. This would help better understand whether patients with 22q11DS tend to have a global deficit in the area of social cognition or whether specific profiles can be defined. Furthermore, little is known about the association between these two processes and psychotic symptoms or social difficulties in this population. One study found that the cognitive dimension of social cognition was significantly associated with parent ratings of social competence [10], but this finding was not replicated in a subsequent study using a different task [13]. Additionally, Jalbrzikowski et al. observed that lower scores on the TASIT were associated with positive symptom severity in patients with 22q11DS but were unrelated to negative symptoms [12]. Finally, clinical and functional correlates of the affective dimension of social cognition have rarely been examined. To date, only one study has examined whether affective ToM contributed to social skills but did not find any significant association [5].

In the present study, the performance of adolescents and young adults with 22q11DS was examined in two tasks measuring affective and cognitive facets of social cognition. A classical emotion recognition paradigm was chosen to measure the affective facet, and a perspective-taking paradigm (an adapted version of the Director task developed by [21]) was used to assess the cognitive facet. The latter task was chosen for its appropriate use across a broad age range, including adulthood. Indeed, it was designed to avoid ceiling effects that are typically observed when adults perform classical ToM or perspective-taking tasks. In line with previous findings in the field of social cognition, we expected patients with 22q11DS to score significantly lower on both tasks compared to a group of typically developing individuals. Given the inter-related nature of the two processes [8], we also expected to observe significant associations between the affective and the cognitive facets in both groups of participants. Finally, in order to improve our knowledge of increased social dysfunction and psychotic symptoms in 22q11DS and typically developing individuals, our last analyses were performed in each group separately. Specifically, we examined whether the performance (indexed by ACC) and/or the efficiency (indexed by RT) to both social cognitive tasks were related to the Schizotypal Personality Questionnaire (SPQ) scores and/or to the Vineland Adaptive Behavior Scales (VABS). Importantly, the literature holds that schizotypal traits assessed at a behavioral level may serve as distal risk marker for psychosis (for a review, see [22]).

## Methods

### Participants

Twenty-nine participants with 22q11DS and 27 typically developing controls (*N* = 5 siblings; *N* = 22 unrelated controls) aged between 11 and 21 years participated in the study. Both groups were matched for age and gender distribution (see Table 1). Some participants met diagnostic criteria for a current DSM-IV disorder according to the Diagnostic Interview for Children and Adolescent (DICA; [23]) for participants below 18 years or the Structured Clinical Interview for DSM-IV (SCID; [24]) diagnoses for adult participants: 12 were diagnosed with an anxiety disorder (specific phobia, generalized anxiety disorder, or social phobia), 6 with a disruptive disorder (ADHD or oppositional defiant disorder), and 2 with a major depressive disorder. None of them met current diagnostic criteria for a psychotic disorder. Eleven (35.5%) participants with 22q11DS were receiving at least one psychotropic medication at the time of testing: 6 were on methylphenidate, 4 on antidepressants, 2 on antipsychotics (because of persistent subthreshold psychotic symptoms), 1 on anxiolytics, 1 on antihistamine, and 1 on anticonvulsant medication. Participants were instructed to take their medication as they usually do during school days. Typically developing controls were screened for the presence of any psychiatric or neurological condition before participating in the study during a phone interview with the parent of the participant. All the potential control participants with a past or present use of psychotropic medication, psychiatric treatment, epilepsy, or any other known neurological condition were excluded. In addition, parents of all control participants completed the Child Behavior Checklist (CBCL; [25]) or the Adult Behavior Checklist (ABCL; [26]). The total problems *t* score was in the non-clinical range for all the controls.

**Table 1:** Descriptive characteristics of participants with 22q11DS and typically developing controls. Unless otherwise specified, mean (SD) are provided

	22q11DS (*N* = 29)	Controls (*N* = 27)	Test
Age	17.79 (2.89)	17.25 (3.34)	*p* = 0.544
Gender (% females)	44.8%	44.4%	*p* = 0.977
Full-scale IQ	75.33 (11.66)	110.73 (13.28)	*p* < 0.001
SPQ cognitive-perceptual	5.29 (5.71)	3.50 (3.36)	*p* = 0.170
SPQ interpersonal	8.34 (5.94)	3.35 (3.75)	*p* = 0.001
SPQ disorganization	4.38 (4.02)	2.80 (3.28)	*p* = 0.127
VABS socialization	77.46 (15.63)	95.89 (11.61)	*p* < 0.001

*SPQ* Schizotypal Personality Questionnaire, *VABS* Vineland Adaptive Behavior Scales

Individuals with 22q11DS were recruited through advertisements in patient association newsletters. The presence of a 22q11.2 microdeletion was confirmed using a quantitative fluorescence polymerase chain reaction (QF-PCR). Typically developing individuals were recruited among the siblings of the participants with 22q11DS or through the local school system.

### Material

#### Intellectual functioning

Intellectual functioning was assessed using the Wechsler Intelligence Scale for Children 3rd edition (WISC-III; [27]) or the Wechsler Adult Intelligence Scale for Adults 3rd edition (WAIS-III; [28]) depending on the age of the participants.

#### Social cognition

The affective facet of social cognition was examined using an emotion recognition computerized paradigm based on the Pictures of Facial Affect by Ekman and Friesen [29]. Eighty black and white photos depicting four basic expressions of facial emotions (happiness, sadness, fear, anger) and 10 neutral expressions were presented to the participants in a randomized order. Each picture was accompanied by five labels (happiness, sadness, fear, anger, calm). Emotional expressions were either mild (50% trials) or intense (50% trials). The participants were instructed to click on the label corresponding to the emotional expression. Any click outside the designated answer areas counted as a no response. The presentation duration of each picture varied as a function of the response speed. In the present analyses, the total number of correct answers (max = 90) and the median reaction time were used.

The cognitive facet of social cognition was evaluated with the French computerized adaptation of the Director Task [21]. Stimuli consisted of 16 slot shelves, eight of them containing eight different objects. The task encompassed two conditions, so called director and no-director, each including experimental, control, and filler trials. In the director condition, a man stood behind the shelf and asked the participant to select and move the objects. Correct answers in the experimental trials required selecting the right object according to the director’s perspective. That is, the participant should choose the object that is visible to the director and ignore the object that is invisible to the director (distractor) even if it fits the instruction best. In control trials, similar objects were arranged in the same order, but an irrelevant object replaced the distractor. In filler trials, instructions only targeted objects seen by the director and the participant. In the no-director condition, the instructions were identical with the exception that they did not come from the director. Indeed, the experimental trials now required the participant to follow a rule that consisted in ignoring objects placed in slots with a gray background. This condition was designed to match the executive processes engaged in the director condition, such as working memory, rule following, or inhibition of the prepotent response. Thus, to sum up, the no-director condition can be considered as a control task that accounts for the cognitive processes beyond perspective-taking involved in the director condition. For a complete description of the task, see [21].

For the purpose of the present study, filler trials were not included in the analyses, and control trials were used for preliminary analyses to test eventual floor or ceiling effects. Main analyses hence involved accuracy (ACC) and response times (RT) for experimental trials in the director and no-director conditions.

#### Clinical measures

In the present study, clinical measures are reported only for patients with 22q11DS. Participants completed the Schizotypal Personality Questionnaire (SPQ; [30]), a 74-item self-report assessing schizotypal experiences that has been validated for use in adolescents and in patients with 22q11DS [2, 31]. The cognitive-perceptual, negative, and disorganization dimensions were used as measures of self-reported schizotypal traits. All the participants were instructed to leave the items blank if they did not understand their meaning. Missing items were reviewed by a member of the research team and filled in with the participants by rephrasing their content if necessary. In addition, the Vineland Adaptive Behavior Scales (VABS; [32]) was also administered to the parents of all participants. The VABS is a semi-structured interview providing information about adaptive skills in children and adolescent in the domains of communication, daily living skills, and socialization. We used the age-appropriate standardized scores (mean = 100; S.D. = 15) of the socialization domain as a measure of social functioning. For participants aged above 18 years, the norms of the upper age level were used, as suggested by the manual. The remaining domains of the VABS (communication and daily living skills) were not examined in the context of this study, as they are conceptually unrelated to social cognition.

### Statistical analyses

Preliminary analyses were performed on the director task to ensure that the difficulty level was suitable for both groups of participants (i.e., test for potential ceiling and floor effects). Potential effects of age and IQ on response time or accuracy scores on the two socio-cognitive tasks were also examined using Pearson correlations. Because difference of IQ is an inherent property of the grouping variable (patients with 22q11DS vs. controls), it is statistically incorrect to control for IQ differences in the group comparisons (see for example [33]).

The main analyses first involved multivariate analyses of variance to compare between-group (22q11DS vs. healthy controls) differences on affective (accuracy and response time from the Emotion Recognition task) and cognitive (accuracy and response time from the Director task) dimensions of social cognition, as well as on levels of schizotypal symptoms (SPQ positive, negative and disorganized dimensions) and social functioning (VABS socialization). Associations, between the accuracy and response time scores of the two socio-cognitive facets were examined using Pearson correlations in participants with 22q11DS and healthy controls separately. Finally, the associations between the accuracy and response time scores of the two socio-cognitive tasks and schizotypal symptoms as well as socialization skills were finally assessed using Spearman correlations.

## Results

### Descriptive characteristics

Descriptive characteristics of both groups are displayed in Table 1. Participants with 22q11DS reported a higher score on the interpersonal dimensions of schizotypal traits (SPQ; *p* = 0.001) than typically developing controls, while similar degrees of disorganized (*p* = 0.127) and cognitive-perceptual (*p* = 0.170) schizotypal traits were observed. Parents also reported significantly worse social functioning in the 22q11DS group compared to the control group (*p* < 0.001). Participants with 22q11DS on medication (any type) did not differ from those not on medication regarding the severity of schizotypal manifestations, full-scale IQ, performance on the emotion recognition task, or performance on the director task (all *p* > 0.05).

### Social cognition

#### Preliminary analyses

Four participants with 22q11DS responded to less than 50% of experimental trials in the director condition. Two participants (one from the control group and one with 22q11DS) scored < 90% of accuracy on the filler trials (in the director and in the non-director conditions). Many factors, such as a lack of motivation and a higher fatigability, may have contributed to the difficulties of those participants who were excluded from the analyses. The final sample was composed of 24 participants with 22q11DS and 26 controls.

Consistent with Dumontheil et al. [21], the suitability of the director task for both groups of participants was preliminarily tested using paired-sample Wilcoxon signed-rank tests. Checks were performed in the control and 22q11DS groups separately. First, the error rate on control trials was compared to the success rate in the experimental trials of the director condition (control group *p* < 0.001; 22q11DS group *p* < 0.01) to verify a significant effect of the condition (i.e., executive versus perspective-taking). Secondly, the correct responses in the experimental trials were compared to the control trials in the no-director condition (*p* < 0.001 in both groups) to make sure that the executive condition was not too easy.

The influence of age and IQ on response times as well as accuracy scores of the two tasks was not statistically significant in any of the two groups (*r* from 0.016 to 0.363, all *p* > 0.05).

#### Between-group differences

A multivariate analysis of variance revealed a significant group difference in socio-cognitive abilities (Pillai’s trace = 0.58, *F* (6.38) = 8.87, *p* < 0.001, partial eta squared = .58). The univariate *F* tests revealed significant group differences in favor of the control group on accuracy scores, both in the director task (in both the director and no-director conditions) and in the emotion recognition task (see Table 2). However, no between-group difference in response times was observed (*p* > 0.05). The results remained similar when participants under antipsychotic medication were excluded from the analyses.

**Table 2:** Means and standard deviations of participants with 22q11DS and typically developing controls for the univariate *F* tests. Response times (RT) are displayed in milliseconds

	22q11DS (*N* = 24)	Controls (*N* = 26)	Test	Effect sizes *partial eta* ^*2*^
Emotion recognition ACC	61.70 (10.74)	70.36 (7.16)	*p* = 0.003	.196
Emotion recognition RT	2193.05 (580.76)	2131.68 (504.50)	*p* = 0.651	.003
Director task—director condition				
Exp trials ACC	0.31 (0.17)	0.59 (0.31)	*p* < 0.001	.241
Exp trials RT	3127.51 (389.63)	3311.39 (318.80)	*p* = 0.066	.066
Director task—no-director condition				
Exp trials ACC	0.46 (0.28)	0.88 (0.15)	*p* < 0.001	.494
Exp trials RT	3310.48 (489.45)	3489.46 (531.48)	*p* = 0.144	.030

*ACC* accuracy, *RT* response time

#### Within-group associations between cognitive and affective facets of social cognition

In the control group, accuracy in the director condition was negatively associated with the response time in the emotion recognition task (*r* = − 0.404, *p* = 0.04). Positive correlations were further observed between accuracy scores in the no-director condition and the emotion recognition task (*r* = 0.532, *p* = 0.006) and between response times in the no-director condition and the emotion recognition task (*r* = 0.586, *p* < 0.001). Following Cohen’s guidelines, these correlations reveal medium (*r* > .30) to large (*r* > .50) effect sizes. The remaining correlations were not significant (all *p* > 0.05). In participants with 22q11DS, no significant association was observed between the two tasks (all *p* > 0.05). This remained unchanged when participants under antipsychotic medication were excluded.

### Within-group associations between social cognition, schizotypal symptoms, and social functioning

In the healthy control sample, the level of positive schizotypal traits was positively associated to response times in the perspective-taking condition of the director task (*r* = 0.561, *p* = 0.004). All the analyses were repeated with the exclusion of siblings from the control group. They all remained unchanged, except that the correlation between response time in the perspective condition of the director task and positive schizotypy became marginally significant (*r* = 0.437, *p* = 0.054).

In participants with 22q11DS, a positive trend was highlighted between the response times on the perspective-taking condition of the director task and levels of negative (*r* = 0.425, *p* = 0.062) and disorganized (*r* = 0.393, *p* = 0.087) schizotypal traits. When participants under antipsychotic medication were excluded from the analyses, these marginal correlations became significant (positive *r* = 0.464, *p* = 0.046; negative *r* = 0.598, *p* = 0.007; disorganized *r* = 0.576, *p* = 0.010). Following Cohen’s guidelines, these correlations reveal medium (*r* > .30) to large (*r* > .50) effect sizes. The remaining correlations were non-significant (all *p* > 0.05).

## Discussion

The present study employed two experimental measures to investigate the cognitive and affective dimensions of social cognition in a sample of individuals with 22q11DS compared to healthy controls. We also examined the extent to which both socio-cognitive dimensions were associated to self-reported schizotypal traits and social functioning. Four main observations summarize the current study: (1) participants with 22q11DS had lower accuracy scores but comparable response times compared to healthy controls on (a) the facial emotion recognition task; (b) the perspective-taking paradigm; (2) the two socio-cognitive dimensions investigated here were correlated in healthy controls; (3) the response time scores in the perspective-taking condition of the director task were associated to positive schizotypal traits in healthy control participants; and (4) the response time scores in the perspective-taking condition of the director task which were marginally associated to negative and disorganized schizotypal traits in participants with 22q11DS. These results will be discussed in relation to the existing body of empirical and conceptual literature.

As expected, individuals with 22q11DS showed lower abilities than healthy controls to correctly recognize facial emotions, which are consistent with previous findings in the literature [5, 10–13]. Atypical behavioral [9, 14, 15] and cerebral [34, 35] processing of faces in 22q11DS, as well as structural alterations in the brain regions involved in facial emotion recognition [36, 37], may contribute to the current results. Indeed, eye-tracking studies have consistently shown different patterns of visual exploration during face-processing tasks involving neutral or emotional stimuli in individuals 22q11DS [14, 15]. Compared to typical and idiopathic developmentally delayed control groups, patients with 22q11DS were shown to spend less time on the eyes and more time on the mouth [15] or the nose [14] when examining faces. Furthermore, abnormal responses to faces have been observed in patients with 22q11DS, which include a lack of normal face selectivity in the fusiform gyrus [34], and a reduced activity compared to controls in brain regions involved in emotion processing during the presentation of diverse emotions at varying intensities [35]. Of note, a recent study reported similar accuracy scores between participants with 22q11DS and healthy controls during an emotion recognition paradigm [14]. The sample and task used by Fanchini et al. may partially explain these contradictory findings. Indeed, emotion recognition was assessed with a dynamic paradigm in a sample of children with 22q11DS, while the data reported here were collected using static stimuli in a group of patients with 22q11DS of a broader age range. Thus, emotion recognition difficulties may appear later in the development and/or be highly influenced by the type of stimuli. Still, these hypotheses remain speculative and should be addressed in further studies that directly compare both tasks in multiple age groups of patients with 22q11DS.

In addition to impairments in the affective dimension of social cognition, participants with 22q11DS struggled to reason according to the perspective of another person in an online communication task, in which they were requested to move objects that can be seen by themselves and their interlocutor. These results are in line with and extend available data on theory of mind (ToM) impairments in this syndrome [10, 12, 13, 18]. This paradigm focuses on a specific component of cognitive ToM (i.e., perspective-taking abilities) that has never been investigated in 22q11DS. It should be noted that although participants with 22q11DS made an increased number of errors compared to the control group, the mean accuracy score in both groups was low. This is consistent with recent findings using the same paradigm in adolescents from the general population that show an improvement of perspective-taking abilities during adolescence and into early adulthood [21]. Impaired performance on this task has been explained by a marked egocentric interference of the self-perspective (i.e., the selection of the correct object from our own perspective and not the one that fits the director’s perspective) [38]. In participants with 22q11DS, perspective-taking abilities might have been influenced by higher-order cognitive difficulties, as perspective-taking was shown to engage working memory or cognitive control processes (e.g., [39]). This interpretation is in line with the fact that participants with 22q11.2DS encountered important difficulties in the no-director condition and that executive functioning represents one of the most affected domains of the 22q11DS phenotype. Indeed, a recent study showed that certain executive functions followed an atypical trajectory and that executive deficits were especially pronounced during adolescence [40]. The association between executive functioning and social cognition has been further pointed out at a cerebral level; the brain regions that sustain executive processes are highly overlapping with the neural networks referred to as the “social brain” [41].

Taken together, the results presented so far suggest that individuals with 22q11DS exhibit difficulties in inferring both cognitive and affective mental states to others, regardless of the type of cue. Indeed, individuals with 22q11DS show similar deficits when they have to attribute an emotional state to someone else based on visible cues (i.e., facial features in the emotion recognition task) and when they have to put themselves in the director’s shoes and use the director’s perspective to resolve the task without manifest clues. In future studies, it might be interesting to disentangle the variables that contribute to the shared variance between the two socio-cognitive processes. Moreover, as social cognition is a complex and multi-dimensional process, forthcoming works are needed to cover other aspects of that higher order ability. We state here that the next step could be, for instance, to replicate the current results with more naturalistic tasks that also extend the range of tested emotions and the modalities of testing (e.g., including auditory processes).

Because two core aspects of the 22q11DS clinical phenotype, namely, schizotypal symptoms and impaired socialization [4, 5], have been related to socio-cognitive impairments in various populations [6] and because these different domains have been tied to overlapping neural networks, our last aim was to examine the associations between these clinical manifestations and the socio-cognitive tasks in both groups of participants. In line with the previous findings [42], participants with 22q11DS were characterized by higher levels of negative symptoms and impaired adaptive social functioning. In the context of this study, the presence of autism spectrum disorder (ASD) or autism traits was not formally assessed. Given that general population studies have shown a significant association between negative schizotypy and autism traits [43, 44], we cannot exclude that the elevated negative schizotypy score observed in the present study would have some link to the potential increased prevalence of patients meeting ASD criteria. However, the question deserves a detailed protocol, using all the appropriate measures that can tackle the issue with respect for its complexity. Similarly, the influence of other clinical diagnoses was not directly investigated. Given the high comorbidity existing between anxiety, mood, and psychotic disorders in 22q11DS [4], this should be addressed in future studies including larger samples. Notwithstanding, we crucially believe that potential associations with other clinical dimensions could inflate the strength of the correlations but would not change our conclusions regarding the present findings.

An association between the degree of schizotypal traits (positive dimension) and the efficiency of perspective-taking processes was observed in typically developing participants, which supports previous broader evidence of social cognition deficits along the psychosis continuum [45, 46]. However, contrary to our hypothesis, schizotypal symptoms and socialization skills were poorly related to the affective and cognitive facets of social cognition in participants with 22q11DS. We were only able to highlight a marginally significant association between slower response times in the perspective-taking task and higher levels of negative and disorganized symptoms, as measured by the SPQ. Of note, associations with all three dimensions of the SPQ became significant when participants under antipsychotic medication were excluded from the analyses.

Regarding the association between schizotypal traits and response times in the director condition, this result was less expected, in light of the absence of difference between the two groups on this variable. This result must be replicated in future studies before any conclusions could be drawn. Yet, statistical and conceptual hypotheses can be put forward to illuminate our data. On the one hand, because the mean accuracy score in the director condition was low, it is probable that RT are more sensitive to individual differences given they have higher variance than ACC scores. On the other hand and given that the RT scores were only calculated for correct items, our result suggests that in PT, the capacity to take the perspective of another person may be less important to consider than the efficiency of that ability. Plausibly, the total variance of socio-cognitive abilities could be explained by partially independent components of speed and accuracy associated to, at least partially, distinct variables. The importance of separating speed and accuracy has been widely proven in intelligence research [47]. To the best of our knowledge, whether a similar latent model might underlie perspective-taking capacities still needs to be established; this should be considered in future studies. Finally, another point that might deserve attention is that social cognitive processes can only fully develop over time. Previous studies have shown that adults are more accurate than youths and children but also tend to take more time (e.g., [21]). Thus, accuracy and response time might be differentially sensitive to typical (e.g., age) and atypical (e.g., schizotypal traits) developmental processes.

Concerning the lack of relation with accuracy scores, a series of prior empirical work has similarly failed to corroborate the theoretical argument that socio-cognitive skills underlie social difficulties and psychiatric symptoms in daily life [48]. Measurement and sample issues may account for the lack of meaningful observations reported here. Indeed, the current study includes experimental tasks that isolate specific socio-cognitive mechanisms rather than encompassing the whole complexity of interpersonal relationships. Of note, both samples included a limited number of participants, implying that some findings might have been significant if tested in bigger groups. As such, measurement limitation may also have contributed to a lack of sufficient power to detect statistically significant within-group associations. Therefore, future work should address the question of cognitive and affective facets of social cognition in 22q11DS in larger samples. Despite these limitations, findings related to response times in the perspective-taking task are promising, especially because this paradigm was designed to bypass the limitations of classical cognitive ToM and perspective-taking measures and is viewed as a better indicator of perspective-taking in real life. Indeed, one of the major criticisms of classical ToM and perspective-taking paradigms is that the participants only play an observer role and have to infer mental content to individuals with whom they are not interacting [49]. “Hence, future studies should replicate these results in a larger sample and attempt to better explain the contribution of perspective-taking impairments in the emergence of psychotic symptoms in 22q11DS, thereby informing future evidence-based treatment”.

## Conclusions

In conclusion, this study provides initial evidence for deficits in two core facets of social cognition in 22q11DS and opens promising research avenues. However, the weak associations observed between the experimental tasks and measures of social functioning or schizotypal symptoms further accentuate the need to better understand the influence of socio-cognitive deficits on the clinical phenotype of patients with 22q11DS.

## Abbreviations

22q11DS: 22q11.2 deletion syndrome
ACC: Accuracy
QF-PCR: Quantitative fluorescence polymerase chain reaction
RT: Response time
SPQ: Schizotypal Personality Questionnaire
TASIT: The Awareness of Social Inference Test
ToM: Theory of mind
VABS: Vineland Adaptive Behavior Scales
